# Numerical investigations of AC arcs’ thermal characteristics in the short gap of copper-cored wires

**DOI:** 10.1038/s41598-024-54911-2

**Published:** 2024-02-20

**Authors:** Yu Li, Rencheng Zhang, Kai Yang, Yufan Qi, Ran Tu

**Affiliations:** Key Laboratory of Process Monitoring and System Optimization for Mechanical and Electrical Equipment, Huaqiao University, Xiamen, 361021 People’s Republic of China

**Keywords:** Plasma physics, Electrical and electronic engineering

## Abstract

Excessive alternating current (AC) arcs generated in electric systems will accumulate heat and easily cause fire. This paper studies the thermal characteristics of different numbers of AC arc plasma generated in a short gap of copper-cored wires in the air. The number of AC arcs is controlled in the AC arc experiment and an infrared thermal imager measures the temperature change at the specified position. Based on magnetohydrodynamics (MHD), a two-dimensional axisymmetric AC arc discharge numerical simulation model is established. The volt-ampere characteristic of the AC arc is used to solve the MHD simulation model to obtain the same 'zero current' characteristics as the real AC arc in the experiment. A large amount of heat accumulates in the electrode gaps when the arc generation, and then the heat dissipates in the 'zero current' stage. The continuously generated arc makes the temperature higher. The volume of the space area with a temperature higher than 10,000 K increases with the arc current, but is unrelated to the number of arcs. The volume of the space area with a temperature higher than 524.15 K and the temperature on the electrode are both positively correlated with the number of AC arcs and arc current. The results of this study can provide a reference for the detection standard of AC arc faults and the prevention of electrical fire.

## Introduction

It can easily cause arc fault when cable aging, loose insulation, grounding fault, improper operation, and man-caused damage appear in the low voltage electricity. Continuous AC arc faults can quickly gather much heat and produce high temperatures. High temperatures carbonize the polyvinyl chloride (PVC) sheath of wires and cause electrical fires^1,2^. The different numbers of AC arc faults have different hazard levels. China and the United States have issued arc fault detection standards based on the number of arc faults^3^. Therefore, it is of great significance to study the thermal characteristics of different numbers of AC arcs generated between short gaps of copper-cored wires.

Arc plasma discharge phenomenon in electrode insulation gap due to a breakdown of the strong electric field. Arc is the coupling result of electric, magnetic, thermal, and flow. Therefore, the study of the arc can obtain numerical simulation results by establishing a multiphysics coupling model. Furthermore, MHD can describe the behavior of conductive fluid in an electromagnetic field so that the MHD model can be used for numerical arc analysis^4^.

The electrode size, spacing, and arc generation position will affect the arc's evolution and the electrical system. MHD models are established to research characteristics of arc under different conditions. For example, arc voltage gradient under electrode spacing^5^, effects of electrode size on a three-phase arc^6^, electrical explosion of the different size copper wires in water^7^, the pit formation on anode surface under the combined action of vacuum arc plasma and electrons^8^, arc plasma expansion in an industrial vacuum arc remelting process^9^, direct current (DC) arc on the photovoltaic power system^10^, arc evolution process in low voltage circuit breakers^11^, arc characteristics in relays at different breaking speeds^12^, characteristics of vacuum arc in anode spot and anode plume^13^, arc reignition in high power relays^14^, nonlinear plasma response and flow evolution in a tokamak^15^. The type of insulating medium in the electrode gap also affects the arc, such as CF_4_ arc plasma^16^, SF_6_ plasma^17^, and lithium plasma^18^. Magnetic field changes will also affect the arc, such as vacuum superconducting plasma jet^19^, AC and DC plasma arcs exposed to cross-fields^20^, and axial magnetic field affects the breaking capacity of DC vacuum circuit breaker and pulsed arcs^21,22^. The MHD model can also be used to study energy conversion, such as the conversion device from mechanical to electrical energy based on MHD^23^. The arc will cause high temperature, and the MHD model can also obtain the temperature field distribution of the arc^24,25^.

In previous work, the arc MHD model was established to research the arc characteristics or the impact of the arc in different conditions. The existing arc MHD models are mostly DC arc, with few AC arc MHD models. The arc is considered pure resistance, so its heat generation power equals the electrical power^26^. In the 'zero current' stage, the electric power of the arc is 0, so no heat is generated. The electric power of the arc in the 'zero current' stage calculated by the existing AC arc MHD model is not 0, so it will bring about errors in heat production. In addition, the number of AC arcs will also affect the generation and accumulation of heat. Therefore, it is of great significance for the MHD model of AC arc to restore the 'zero current' characteristics of real AC arcs and to study the thermal characteristics of different numbers of AC arcs.

This paper uses copper-cored wires as the discharge electrode to generate AC arcs and conducts AC arc experiments with a controllable number of AC arcs. The AC arcs generated between the short gap of copper-cored wires in the air and the temperature at the specified position are measured. The AC arc MHD model established by COMSOL Multiphysics commercial simulation software, and the thermal characteristics of AC arcs with different numbers and currents are studied. The experimental results are compared with the calculation results of the AC arc MHD model, and the AC arc MHD model obtains the temperature change in the electrode gap, the spatial temperature distribution characteristics, and the temperature on the electrode.

## Experiments

### Experimental setup

**Figure 1:**
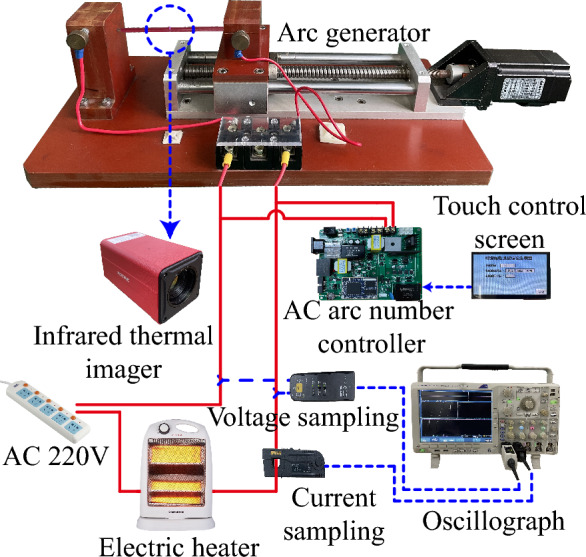
AC arc experimental device platform.

Figure 1 shows the AC arc experimental device platform. The copper-cored wire on the left side is a fixed electrode, and the right side is a movable electrode. The gap between the electrodes of the arc generator device is adjustable. The moving electrode is far away from the fixed electrode to produce a short gap, and an AC arc will occur at an appropriate gap distance. The outer skin of copper-cored wire is PVC, the thickness of PVC is 0.62 mm, the radius of the copper core is 1.38 mm, and the cutting length of the copper core end is 1 mm. The load is a resistive electric heater with adjustable heating power. The Keysight N2790A high-voltage differential probe collects the AC arc voltage, and the Keysight N2781B hall current sensor collects the AC arc current. The Agilent technologies DSO7104B oscilloscope displays and stores the arc voltage and current. The FOTRIC 600C infrared thermal imager measures the temperature distribution on the outer skin of the copper-cored wire. The entire experimental device is placed in a dark box for experiments.

**Figure 2:**
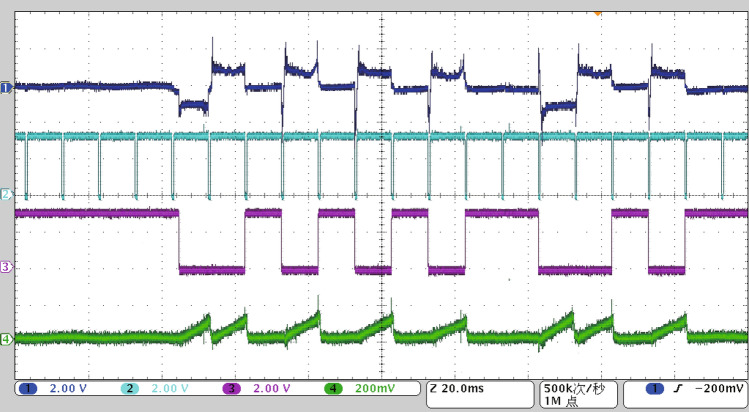
The primary signal timing of the controllable arc auxiliary device on the oscillograph.

Since the number of AC arcs generated in the short gap by directly using the arc generator is uncertain, an auxiliary device named the AC arc number controller needs to be connected at both ends of the electrode, and the number of AC arcs should be input through the touch screen. The working principle of the controllable arc auxiliary device is as follows. Firstly, the zero-crossing time of the AC is detected, and the two adjacent zero-crossing times determine an AC half-wave. Then, in an AC half-wave, the IGBT connected in parallel at both ends of the electrode is controlled to conduct. The current flows through the IGBT, so there is no arc. On the contrary, the control IGBT is disconnected, and the arc generator will generate an arc. Therefore, the connection and disconnection of IGBT in the specified AC half-wave are controlled to generate a controllable number of AC arcs. Figure 2 is the timing diagram of the primary signal of the controllable arc auxiliary device. Signal 2 is an AC zero-crossing signal. When the AC voltage is near the zero-crossing point, signal 2 outputs a low level. There is an AC half-wave between two adjacent falling edges of signal 2. Signal 3 is the IGBT control signal. IGBT closed when signal 3 outputs a high level. Signal 1 is the voltage signal at both ends of the electrode collected by the high-voltage probe. When the IGBT is closed, signal 1 collects the voltage drop at both ends of the IGBT. When the IGBT is disconnected, signal 1 collects the AC arc voltage generated by the arc generator. Signal 4 is the arc half-wave voltage integral signal. In one arc half-wave time, the absolute value of signal 1 is integrated. Through the integral value of the output of signal 4, it can be judged whether the AC arc is really generated in the specified AC half-wave. Therefore, the arc generator can generate a specified number of AC arcs through the controllable arc auxiliary generating device.

Table 1 shows the experimental conditions. The peak value of the arc current ${I}_{P}$ is adjusted to about 2/4/6 A by the electric heater. The electrode gap distance can be adjusted to 0.25 mm. A controllable arc auxiliary generator is used to control the generation of 1 to 10 continuous AC arcs between the electrode gaps. The alternating change of the anode and cathode in different half-waves of the AC arc. In order to facilitate the analysis of subsequent experimental results, it is considered that the arc current in the first AC half-wave time in the experiment flows from the fixed electrode to the moving electrode, which is considered an effective experiment.

**Table 1:** The conditions of experiments.

Parameters	Value
Supply voltage/(V)	AC 220
Frequency/(Hz)	50
Peak current (${I}_{P}$)/(A)	2/4/6
Electrode gap distance (*d*)/(mm)	0.25
Number of AC arcs (*n*)	1/2/3/4/5/6/7/8/9/10
Ambient temperature/K	293.15 ± 2
Humidity/%	50 ± 5

### Volt-ampere characteristics of AC arc

**Figure 3:**
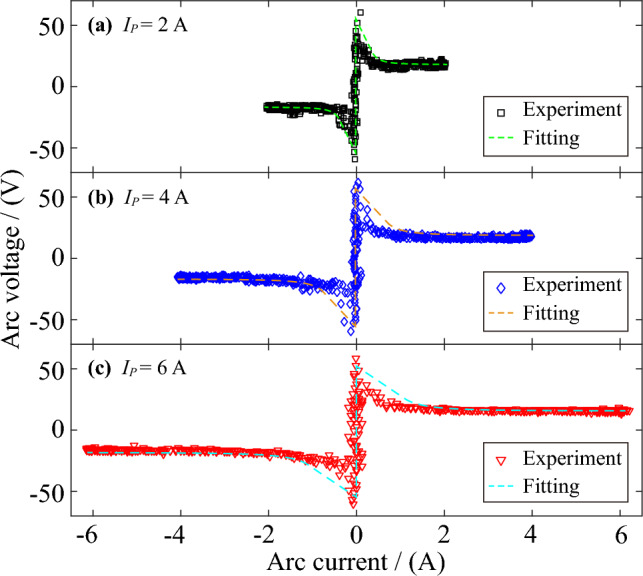
Volt-ampere characteristics of the AC arc.

According to the arc voltage and arc current collected by the AC arc experiment, the Volt-ampere characteristic relationships of the AC arc are obtained in Fig. 3. In the experiment, the electrode gap is broken down to generate an arc when the voltage at both ends of the electrode rises to 48.5–64.7 V. Therefore, the breakdown voltage difference makes the AC arc's breakdown time different. The maximum breakdown time difference accounts for about 1.65% of an AC half-wave time, so the breakdown time difference has little effect on the arc burning time. In order to facilitate the calculation of the MHD numerical simulation model, the average breakdown voltage of 58.3 V is selected, so the opening time of the arc in a half wave ${t}_{on}$ is set to 0.0006 s. Each AC arc's half-wave breakdown and extinction time are approximately symmetrical about the peak time (0.005 s), so the extinction time ${t}_{off}$ is set to 0.0094 s.

After the breakdown of the electrode gap, a stable combustion AC arc is generated. It can be seen from Fig. 3 that the voltage of the AC arc during stable combustion does not change with the change of arc current. The arc voltage mainly includes anode voltage, arc column voltage, and cathode voltage. It is generally believed that the arc length of the long arc is greater than the arc diameter and vice versa. The voltage of the long arc is mainly affected by the arc column voltage, and the voltage of the short arc is primarily affected by the anode voltage and the cathode voltage. The arc studied in this paper belongs to the short arc, so the arc column voltage is not considered^27^. The anode voltage drop and cathode voltage drop are related to the electrode material and remain unchanged. Therefore, the AC arc voltage remains unchanged when the AC arc is in stable combustion.

The voltage and current of the AC arc obtained by the experiment are fitted. Equations (1), (2) are the fitting formulas of the first half-time in an AC half-wave. It is considered that the voltage and current of the arc are symmetrical about the AC arc current peak time in an AC half-wave, and the arc voltage and current in the adjacent alternating current half-wave are opposite. When the arc voltage is less than the arc opening time, the circuit is in an open state, so the voltage is the AC power supply voltage, and the arc current is 0. The voltage change of the AC arc is small when it is stably generated, and the stable arc voltage is kept between 18 and 20 V. The arc current can be fitted as a sinusoidal function of the peak current as ${I}_{P}$. The voltage-current characteristics image of the AC arc after fitting is shown in Fig. 3. After fitting, *K*_*1*_ is 1.76 × 10^7^, *K*_*2*_ is − 2.403, *K*_*3*_ is 18.38. $t$ is time. The Eqs. (1), (2) will also be applied as current density to the solution of the subsequent MHD model.

1 $${U}_{arc}=\left\{\begin{array}{cc}220\times \sqrt{2}\times {\text{sin}}(100\times \pi \times t)& 0\le t\le {t}_{on}\\ {K}_{1}\times {t}^{{K}_{2}}+{K}_{3}& {t}_{on}<t\le 0.005\end{array}\right.$$

2 $${I}_{arc}=\left\{\begin{array}{cc}0& 0\le t\le {t}_{on}\\ {I}_{P}\times {\text{sin}}(100\times \pi \times t)& {t}_{on}<t\le 0.005\end{array}\right.$$

## Models

### Geometric model and material parameters

**Figure 4:**
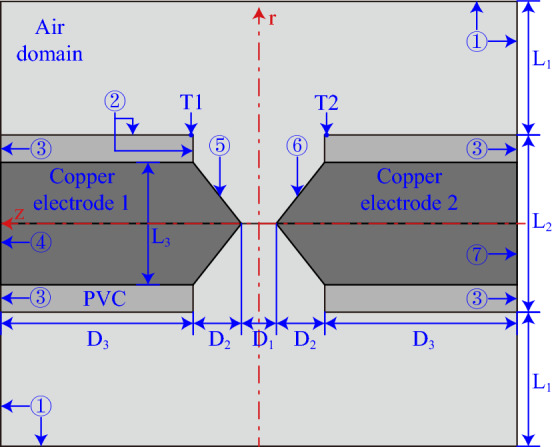
The cross-section of the geometric model.

A two-dimensional axisymmetric geometric model is established for the AC arc MHD model. Figure 4 shows the section of the geometric model and corresponds to the experiment in Fig. 1. Boundary 4 and boundary 7 are connected in series to the AC circuit, and an AC arc is generated between the copper electrodes. The electrode gap length D_1_ changed to 0.25 mm. The end section in the copper-cored wire is sharpened and D_2_ is 1 mm.

**Figure 5:**
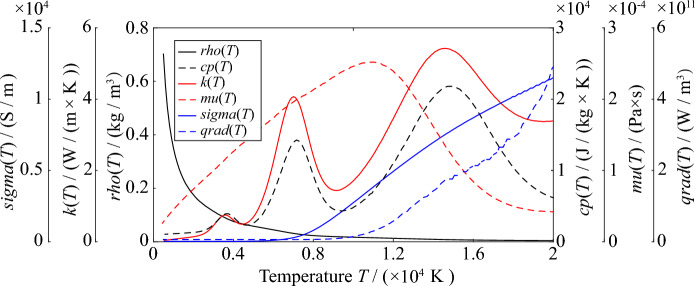
The relationship between air physical parameters and temperature.

Table 2 shows the physical parameters of copper and PVC in the geometric model. Copper and PVC are solids, so the physical parameters of copper and PVC used in the MHD numerical model calculation are constant. The relationship between air density (*rho*), constant pressure heat capacity (*cp*), dynamic viscosity (*mu*), thermal conductivity (*k*), total cumulative radiation coefficient (*qrad*), electrical conductivity (*sigma*) and temperature (*T*), respectively, are shown in Fig. 5^25^.

**Table 2:** Material parameters in geometric model.

Parameters	Value
Conductivity of copper/(S/m)	5.998 × 10^7^
Heat capacity at constant pressure of copper/(J/kg/K)	385
Density of copper/(kg/m^3^)	8940
Thermal conductivity of copper/(W/m/K)	400
Surface emissivity of copper	0.5
Conductivity of PVC/(S/m)	1.0 × 10^–4^
Heat capacity at constant pressure of PVC/(J/kg/K)	1000
Density of PVC/(kg/m^3^)	500
Thermal conductivity of PVC/(W/m/K)	0.16
Surface emissivity of PVC	0.8

### MHD model equations

The AC arc MHD model involves coupling multiple physical fields. Therefore, to reduce the complexity of the model and the faster convergence of numerical calculation, some assumptions are introduced in the MHD model. The characteristic scale and characteristic time of air arc plasma are much larger than the average free path and average collision time of particles, so it is not necessary to consider the motion of a single particle. This paper considers the AC arc as a continuous medium in a local thermodynamic equilibrium state and regards the AC arc as a continuous medium. The arc plasma is a stable, compressible fluid, and the flow of the arc plasma is laminar. The arc burning time is very short, and the arc current is small. The concentration of the electrode vapor is much smaller than the concentration of the air, so the evaporation of the electrode material and the erosion of the electrode contact by the arc are not considered.

The arc plasma generated by the electrode gap in Fig. 4 satisfies the mass conservation equation, momentum conservation equation, energy conservation equation, Ohm's law, Faraday's law, and Ampere's law^27–30^.

The air arc plasma belongs to the conductive fluid, and its mass remains unchanged during the flow of the fluid. Therefore, the mass conservation of the arc plasma is described as Eq. (3). The momentum change rate of the arc plasma is the sum of the forces acting on its material. Therefore, the momentum conservation equation of arc plasma in $r$, $\theta$ and $z$ direction can be expressed as Eq. (4). In the arc plasma flow field, The volume force of the fluid element mainly includes the Lorentz force, gravity, and electric force. Due to the electrical neutrality of the plasma, the electric force is much smaller than the Lorentz force. The influence of gravity is also minimal. Therefore, the volume force mainly considers the Lorentz force. ${F}_{r}$, ${F}_{\theta }$, ${F}_{z}$ are the component vectors of ${\varvec{F}}$(total volume force of fluid), and can be expressed as Eq. (5).

3 $$\frac{\partial \rho }{\partial t}+\frac{1}{r}\frac{\partial \left(\rho r{v}_{r}\right)}{\partial r}+\frac{1}{r}\frac{\partial \left(\rho {v}_{\theta }\right)}{\partial \theta }+\frac{\partial \left(\rho {v}_{z}\right)}{\partial z}=0,$$

4 $$\left\{\begin{array}{c}\rho \left(\frac{d{v}_{r}}{dt}-\frac{{v}_{\theta }^{2}}{r}\right)=\rho {F}_{r}+\frac{1}{r}\left[\frac{\partial \left(r{p}_{rr}\right)}{\partial r}+\frac{\partial {p}_{r\theta }}{\partial \theta }+\frac{\partial \left(r{p}_{zr}\right)}{\partial z}\right]-\frac{{p}_{\theta \theta }}{r}\\ \rho \left(\frac{d{v}_{\theta }}{dt}+\frac{{v}_{r}{v}_{\theta }}{r}\right)=\rho {F}_{\theta }+\frac{1}{r}\left[\frac{\partial \left(r{p}_{r\theta }\right)}{\partial r}+\frac{\partial {p}_{\theta \theta }}{\partial \theta }+\frac{\partial \left(r{p}_{\theta z}\right)}{\partial z}\right]+\frac{{p}_{r\theta }}{r}\\ \rho \frac{d{v}_{z}}{dt}=\rho {F}_{z}+\frac{1}{r}\left[\frac{\partial \left(r{p}_{zr}\right)}{\partial r}+\frac{\partial {p}_{\theta z}}{\partial \theta }+\frac{\partial \left(r{p}_{zz}\right)}{\partial z}\right]\end{array}\right.,$$

5 $$\left\{\begin{array}{c}{F}_{r}={J}_{\theta }{B}_{z}-{J}_{z}{B}_{\theta }\\ {F}_{\theta }={J}_{z}{B}_{r}-{J}_{r}{B}_{z}\\ {F}_{z}={J}_{r}{B}_{\theta }-{J}_{\theta }{B}_{z}\end{array}\right.,$$

6 $$\left\{\begin{array}{c}{p}_{rr}=-p+2\mu \frac{\partial {v}_{r}}{\partial r}+\left({\mu }^{\mathrm{^{\prime}}}-\frac{2}{3}\mu \right)\nabla \cdot v\\ {p}_{\theta \theta }=-p+2\mu \left(\frac{1}{r}\frac{\partial {v}_{\theta }}{\partial \theta }+\frac{{v}_{r}}{r}\right)+\left(\mu \mathrm{^{\prime}}-\frac{2}{3}\mu \right)\nabla \cdot v\\ {p}_{zz}=-p+2\mu \frac{\partial {v}_{z}}{\partial z}+\left({\mu }^{\mathrm{^{\prime}}}-\frac{2}{3}\mu \right)\nabla \cdot v\end{array},\right.$$

7 $$\left\{\begin{array}{c}{{p}_{\theta r}=p}_{r\theta }=\mu \left(\frac{\partial {v}_{\theta }}{\partial r}+\frac{1}{r}\frac{\partial {v}_{r}}{\partial \theta }-\frac{{v}_{\theta }}{r}\right)\\ {p}_{\theta z}={p}_{z\theta }=\mu \left(\frac{1}{r}\frac{\partial {v}_{z}}{\partial \theta }+\frac{\partial {v}_{\theta }}{\partial z}\right)\\ {p}_{zr}={p}_{rz}=\mu \left(\frac{\partial {v}_{r}}{\partial z}+\frac{\partial {v}_{z}}{\partial r}\right)\end{array},\right.$$

8 $$\nabla \cdot {\varvec{v}}=\frac{1}{r}\frac{\partial \left(r{v}_{r}\right)}{\partial r}+\frac{1}{r}\frac{\partial {v}_{\theta }}{\partial \theta }+\frac{1}{r}\frac{\partial \left(r{v}_{z}\right)}{\partial z},$$

where $\rho$ is arc plasma density. ${v}_{r}$, ${v}_{\theta }$, ${v}_{z}$ are the component vectors of ${\varvec{V}}$ (flow velocity of arc plasma) in the cylindrical coordinate system. ${p}_{rr}$, ${p}_{\theta \theta }$, ${p}_{zz}$ are vertical stress respectively in Eq. (6). ${p}_{r\theta }$, ${p}_{\theta r}$, ${p}_{\theta z}$, ${p}_{z\theta }$, ${p}_{rz}$, ${p}_{zr}$ are circumferential stress respectively in Eq. (7). ${J}_{r}$, ${J}_{\theta }$, ${J}_{z}$ are the component vectors of ${\varvec{J}}$ (current density) in $r$, $\theta$ and $z$ directions. ${B}_{r}$, ${B}_{\theta }$, ${B}_{z}$ are the component vectors of ${\varvec{B}}$ (Magnetic inductive) in $r$, $\theta$ and $z$ directions. $p$ is the pressure of arc plasma. $\mu$ is the viscous coefficient. $\mu {\prime}$ is second viscous coefficient. $\nabla$ is the hamiltonian operator and $\nabla \cdot {\varvec{v}}$ can be expressed as Eq. (8).

Arc plasma satisfies energy conservation. The change rate of the total energy of the finite volume element is equal to the sum of the power of the force acting on the volume element and the heat transmitted through the surface in unit time. Therefore, the mechanical energy dissipated by the viscous arc plasma discussed in this paper is wholly converted into internal energy. The arc can be regarded as a pure resistance, so its heat production mainly considers Joule heat. The arc plasma energy conservation equation expressed by entropy is shown in Eq. (9). The energy dissipation function is Eq. (10). AC arc plasma is regarded as gas, which satisfies the gas state equation as shown in Eq. (11).

9 $$\rho T\frac{ds}{dt}=\Phi -\frac{p}{r}\left(\frac{\partial \left(r{v}_{r}\right)}{\partial r}+\frac{\partial {v}_{\theta }}{\partial \theta }+\frac{\partial \left(r{v}_{z}\right)}{\partial z}\right)+{\lambda }_{T}\left(\left[\frac{1}{r}\frac{\partial }{\partial r}\left(r\frac{\partial T}{\partial r}\right)+\frac{1}{{r}^{2}}\frac{{\partial }^{2}T}{\partial {\theta }^{2}}+\frac{{\partial }^{2}T}{\partial {z}^{2}}\right]\right)+\frac{1}{\sigma }\left({J}_{r}^{2}+{J}_{\theta }^{2}+{J}_{z}^{2}\right)-\varepsilon {\sigma }_{s}\left({T}^{4}-{T}_{0}^{4}\right),$$

10 $$\Phi =-\frac{2}{3}\mu {\left[\frac{1}{r}\frac{\partial \left(r{v}_{r}\right)}{\partial r}+\frac{1}{r}\frac{\partial {v}_{\theta }}{\partial \theta }+\frac{1}{r}\frac{\partial \left(r{v}_{z}\right)}{\partial z}\right]}^{2}+2\mu \left[{\left(\frac{\partial {v}_{r}}{\partial r}\right)}^{2}+{\left(\frac{\partial {v}_{\theta }}{\partial \theta }\right)}^{2}+{\left(\frac{\partial {v}_{z}}{\partial z}\right)}^{2}\right]+\mu \left[{\left(\frac{1}{r}\frac{\partial {v}_{z}}{\partial \theta }+\frac{\partial {v}_{\theta }}{\partial z}\right)}^{2}+{\left(\frac{\partial {v}_{r}}{\partial z}+\frac{\partial {v}_{z}}{\partial r}\right)}^{2}+{\left(\frac{\partial {v}_{\theta }}{\partial r}+\frac{1}{r}\frac{\partial {v}_{r}}{\partial \theta }\right)}^{2}\right],$$

11 $$p={\text{R}}\rho T,$$

where ${\text{s}}$ is entropy. $\Phi$ is dissipation item. ${\lambda }_{T}$ is thermal conductivity. $\sigma$ is electric conductivity. $\varepsilon$ is surface emissivity. ${\sigma }_{s}$ is Steffen-Boltzmann constant. ${T}_{0}$ is ambient temperature. ${\text{R}}$ is gas constant.

The arc plasma belongs to the conductor, so a series of electromagnetic field equations are satisfied during the arc generation. Ohm's laws are expressed as Eq. (12). Faraday's laws are expressed as Eq. (13). Ampere's laws are expressed as Eq. (14). The current intensity and magnetic induction intensity are field without source, so $\nabla \cdot {\varvec{J}}$ and $\nabla \cdot {\varvec{B}}$ are both equal to 0, expressed as Eq. (15).

12 $$\left\{\begin{array}{c}{J}_{r}=\sigma \left({E}_{r}+{V}_{\theta }{B}_{z}-{V}_{z}{B}_{\theta }\right)\\ {J}_{\theta }=\sigma \left({E}_{\theta }+{V}_{z}{B}_{r}-{V}_{r}{B}_{z}\right)\\ {J}_{z}=\sigma \left({E}_{z}+{V}_{r}{B}_{\theta }-{V}_{\theta }{B}_{r}\right)\end{array},\right.$$

13 $$\left\{\begin{array}{c}\frac{1}{r}\frac{\partial {E}_{z}}{\partial \theta }-\frac{\partial {E}_{\theta }}{\partial z}=-\frac{\partial {B}_{r}}{\partial t}\\ \frac{\partial {E}_{r}}{\partial z}-\frac{\partial {E}_{z}}{\partial r}=-\frac{\partial {B}_{\theta }}{\partial t}\\ \frac{\partial {E}_{\theta }}{\partial r}-\frac{1}{r}\frac{\partial {E}_{r}}{\partial z}=-\frac{\partial {B}_{z}}{\partial t}\end{array},\right.$$

14 $$\left\{\begin{array}{c}\frac{1}{r}\frac{\partial {B}_{z}}{\partial \theta }-\frac{\partial {B}_{\theta }}{\partial z}={\mu }_{0}{J}_{r}\\ \frac{\partial {B}_{r}}{\partial z}-\frac{\partial {B}_{z}}{\partial r}={\mu }_{0}{J}_{\theta }\\ \frac{\partial {B}_{\theta }}{\partial r}-\frac{1}{r}\frac{\partial {B}_{r}}{\partial z}={\mu }_{0}{J}_{z}\end{array},\right.$$

15 $$\left\{\begin{array}{c}\nabla \cdot {\varvec{J}}=0\\ \nabla \cdot {\varvec{B}}=0\end{array},\right.$$

where ${E}_{r}$, ${E}_{\theta }$, and ${E}_{z}$ are the component vectors of $E$ (electric field) in $r$, $\theta$, and $z$ directions. ${\mu }_{0}$ is magnetic permeability.

When the arc is generated, much Joule heat accumulates between the electrode gaps. The Joule heat in the arc area conducts heat to the electrode and air which can be expressed as Eq. (16). Ignoring the weak Joule heat of the electrode itself, the Joule heat is equal to 0 outside the arc region and after the arc is extinguished. In addition, convective heat transfer and radiative heat transfer are also occurring on the electrode surface, which can be expressed as Eq. (17).

16 $$\frac{\partial \left({\rho }_{m}{c}_{pm}{T}_{m}\right)}{\partial t}-\nabla \cdot \left({k}_{m}\nabla {T}_{m}\right)=Q,$$

17 $${Q}_{sur}=h\left({T}_{sur}-{T}_{0}\right)+{\varepsilon }_{sur}{\sigma }_{s}\left({T}_{sur}^{4}-{T}_{0}^{4}\right),$$

where ${\rho }_{m}$ is density, ${c}_{pm}$ is heat capacity at constant pressure, ${k}_{m}$ is thermal conductivity, ${T}_{m}$ is material temperature, ${\text{h}}$ is coefficient of convective heat transfer, ${T}_{sur}$ is electrode surface temperature, ${\varepsilon }_{sur}$ is material surface emissivity. The above physical parameters are related to the properties of the material itself.

### Boundary conditions

The initial conditions and boundary conditions of the geometric model in Fig. 4 need to be set. The initial temperature of the MHD model is 293.15 K, which is consistent with the experiments. The initial potential of the computational domain of the whole MHD model is 0 V and satisfies the charge conservation and Ampere's law. The magnetic vector potential is 0 Wb/m. The initial atmospheric pressure is 1 atm, and the initial velocity is 0 m/s.

The boundary conditions corresponding to the geometric model of the MHD model in this paper are set as shown in Table 3. In addition, the electric potential of boundary 7 is set to 0 V, the current flows from boundary 4 and flows out from boundary 7. An arc is generated between boundary 5 and boundary 6.

**Table 3:** Boundary conditions.

Boundary	Temperature, *T* (K)	Pressure *p* (atm)	Flow velocity, *v* (m/s)	Magnetic vector potential, *A* (Wb/m)
1	$\frac{\partial T}{\partial n}=0$	1	$\frac{\partial v}{\partial n}=0$	0
2, 5, 6	Equation (17)	$\frac{\partial p}{\partial n}=0$	0	$\frac{\partial A}{\partial n}=0$
3, 4, 7	*T* = *T*_*0*_	$\frac{\partial p}{\partial n}=0$	0	$\frac{\partial A}{\partial n}=0$

## Results

### AC arc voltage and current

**Figure 6:**
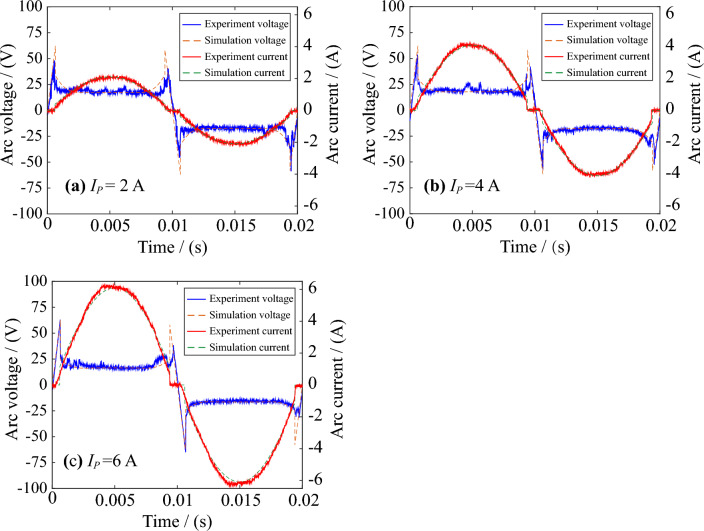
Comparisons of AC arc voltage and current obtained by MHD numerical models and experiments.

The arc voltage and arc current obtained by experiments and MHD models are compared in Fig. 6. The difference between the arc voltage of the MHD model and the real AC arc voltage obtained by the experiment is slight. Before the electrode gap is broken down, the voltage is 220 V AC power supply. After the electrode gap is broken down, the arc burns stably, and the arc voltage remains approximately stable. The current is 0 before the electrode gap is broken down. When the arc burns stably, the AC arc current approximately changes sinusoidally^31^. The arc is often regarded as a nonlinear pure resistance, so the heat generated by the arc is joule heat^27^. It can be seen from Fig. 6 that the AC arc voltage and current of the MHD model are close to the real arc, so the AC arc thermal characteristics obtained by the MHD model will also be close to the real arc in the experiment.

### Temperature changes in the gap and surrounding space

**Figure 7:**
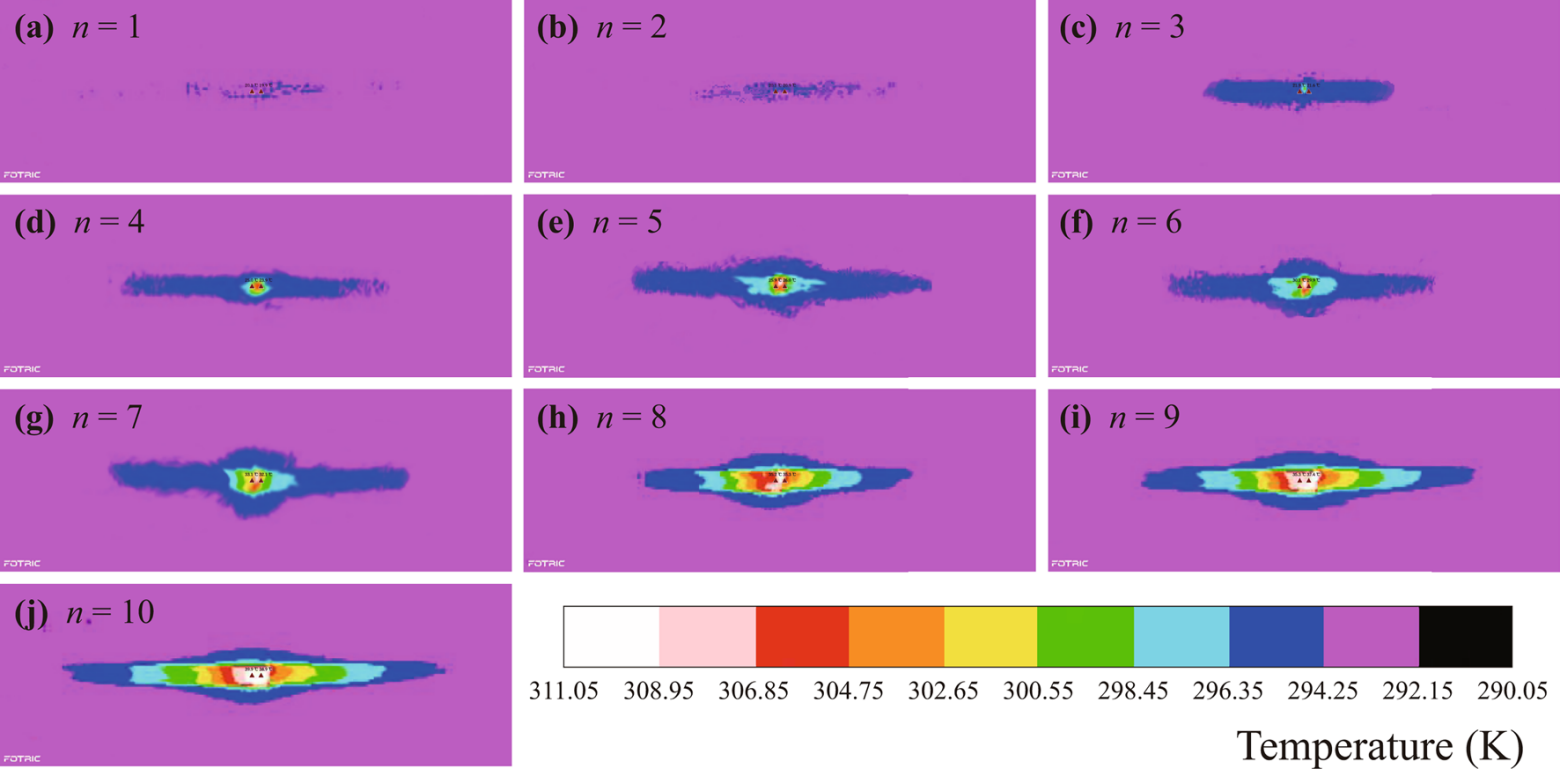
The infrared thermal imager obtains the temperature distribution of *n* AC arcs generated continuously when ${I}_{P}=2\mathrm{ A}$.

Since the temperature of the AC arc plasma is far beyond the range of the infrared thermal imager, the temperature of the arc combustion cannot be directly photographed. Therefore, after continuously *n* AC arcs are extinguished, the infrared thermal imager is triggered to shoot. Figure 7 shows examples of the temperature distribution after generating 1 to 10 AC arcs when ${I}_{P}=2\mathrm{ A}$. It can be seen that with the increase in the number of AC arcs, the temperature is rising, and the temperature near the gap is higher than that far away from the gap.

**Figure 8:**
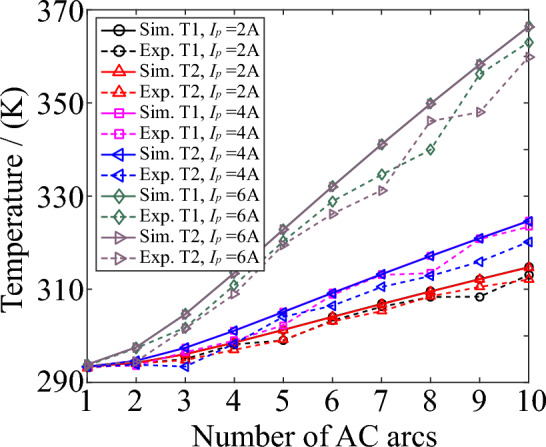
The comparison in the temperature of T1 and T2 obtained by the MHD simulation model and infrared thermal imager.

The infrared thermal imager sets two temperature measuring points, T1 and T2. The diameter of the temperature measuring point is less than 1 mm. They are placed at the discharge ends of the fixed and moving electrodes to measure the temperature of the PVC outer skin of the copper-cored wire. At the same time, the locations of the temperature measurement points in the MHD model are shown in Fig. 4. Figure 8 shows the temperature comparison of T1 and T2 in the MHD model and experiment. With the increase of the number of AC arcs and the increase of arc current, the temperature of T1 and T2 will increase. The MHD model is in an ideal state, so the temperatures of T1 and T2 obtained by the MHD model are consistent. The T1 and T2 temperatures obtained in the experiment are slightly smaller than the MHD model. The uncertainties in the experiment cause this error. For example, the experiment cannot completely control the initial windless environment. The overall relative error increases with the number of AC arcs, and the relative error remains within 8%. Therefore, the MHD model is more accurate in solving the thermal characteristics of 1 to 10 AC arcs.

**Figure 9:**
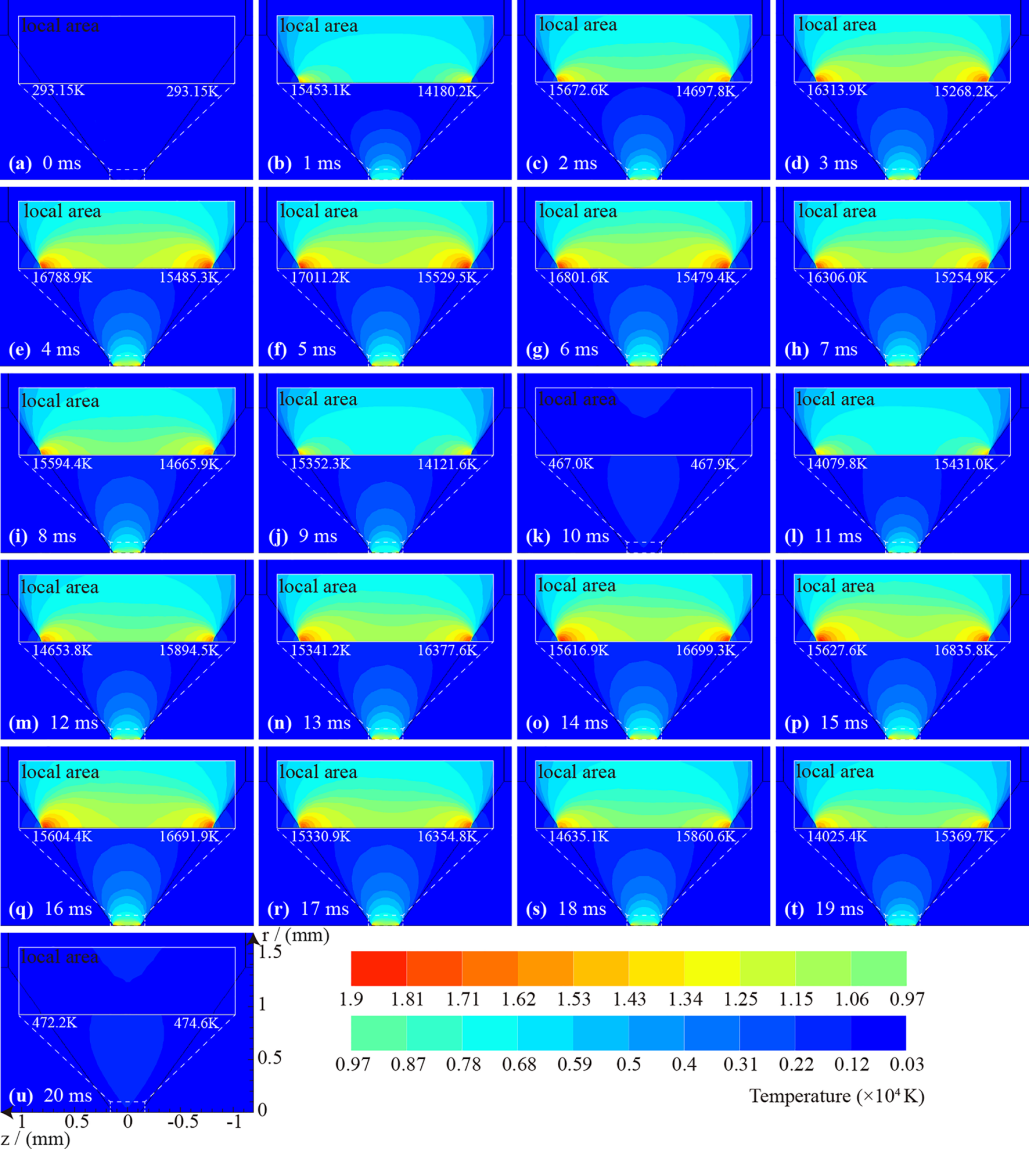
The temperature distribution of 2 AC arcs generated in the gap when ${I}_{P}=2\mathrm{ A}$.

The case in Fig. 9 shows the temperature distribution of 2 AC arcs generated in the gap when ${I}_{P}=2\mathrm{ A}$. At 0 ms, no arc has been generated, and the temperature is 293.15 K. At 1 ms, an arc has been generated, and the temperature in the electrode gaps rises rapidly. The temperature in the central region of the gap can be higher than 10,000 K. The highest temperatures in the gap near the anode and cathode are 15,352.3 K and 14,121.6 K, so the temperature near the anode is higher. At 1 ms to 5 ms, the temperature in the gap continues to rise, and the heat diffuses around and causes the surrounding temperature to rise. At 5 ms, the arc current is at the peak, and the heat production is the highest. After the peak of 5 ms, the temperature in the gap gradually decreases, but the heat still spreads around. After that, the arc enters the 'zero current' stage, and the arc is extinguished and no longer produces heat. For example, the heat in the electrode gap dissipates rapidly at 10 ms, but there is still some afterheat.

After the first arc is extinguished, if the arc is no longer generated, the heat will gradually dissipate. If the arc continues to be generated, the next arc will be generated on the afterheat of the previous arc. After 10 ms, it enters the second AC arc. The anode and cathode are opposite to the previous arc. The maximum temperature near the electrode of the second AC arc is consistent with the previous one, and both increase before the peak time and decrease after the peak time. At 20 ms, the residual temperature after the second AC arc is extinguished is higher than the first arc.

**Figure 10:**
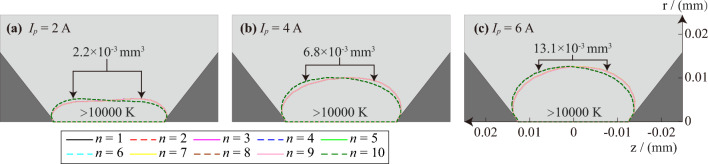
The boundary of the area where the temperature is higher than 10,000 K at the peak time of the *n*th AC arc.

This paper pays special attention to the size of the area where the temperature is higher than 10,000 K at each AC half-wave peak time and names it as the high-temperature area of the AC arc. Figure 10 shows the range of the high-temperature area at the peak time of the *n*th AC arc. When *n* changes from odd to even, the anode and cathode of the AC arc will be interchanged, resulting in the boundary range of the high-temperature area of the AC arc being related to the number of AC arcs being odd or even. However, the volume of the high-temperature area of the AC arc does not change when the AC arc current is the same. It can be found from Fig. 10 that the volume of the high-temperature AC arc area increases with the arc current.

**Figure 11:**
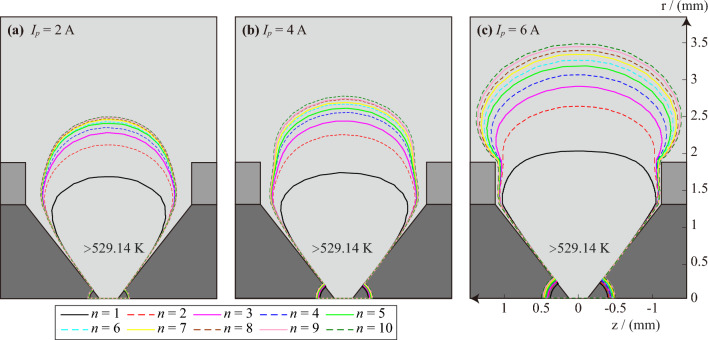
The boundary range of the area where the temperature is higher than 529.14 K at the peak time of the *n*th AC arc.

**Figure 12:**
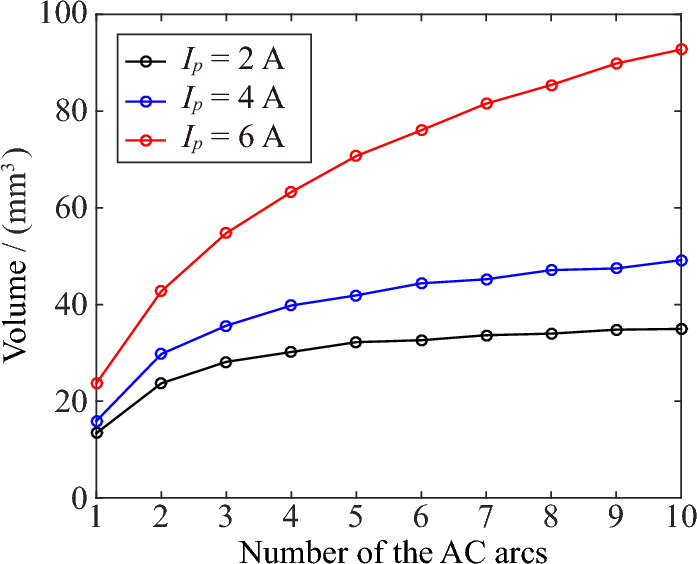
The volume of the fire hazardous area of the AC arc.

Since the discharge electrode used in this paper is copper-cored wire which outer skin is PVC, the burning point of PVC is 529.14 K. Therefore, the continuous generation of AC arc will increase the temperature of PVC and easily ignite PVC to produce electrical fire. This paper also pays special attention to the size of the area where the temperature is higher than 529.14 K and names it as fire hazardous area. Figures 11 and 12 are the boundary range and volume of the fire hazardous area at the peak time of the *n*th AC arc, respectively. The continuous AC arc will accumulate more heat, so the size of the fire danger zone will increase with the number of AC arcs.

The electric power of the arc is the multiplication of the arc current and the arc voltage. The arc voltage in the short arc is unchanged, so the larger arc current will produce greater arc power. The arc is regarded as a pure resistance, so a larger arc current will also bring more heat, and the volume of the fire danger zone will increase.

### Temperature changes on the electrode

**Figure 13:**
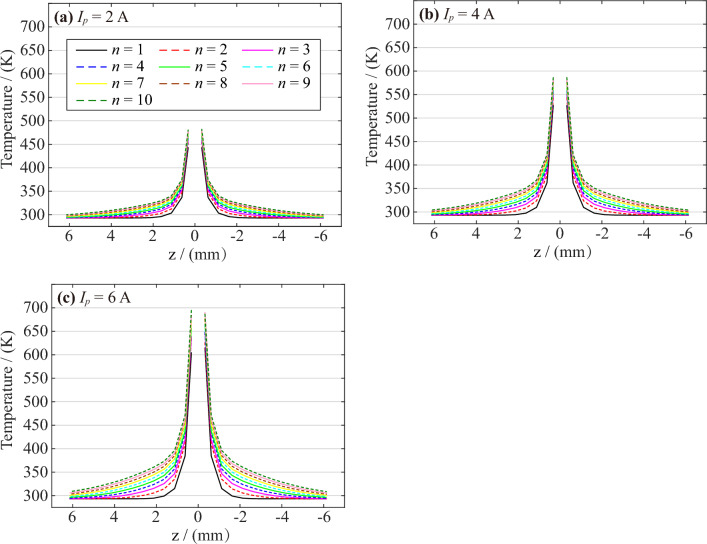
Temperature distribution of the electrode center at the peak time of the nth AC arc.

The high-temperature arc will also dissipate heat to the copper core through heat conduction, so the temperature on the copper-cored electrode will also increase. Figure 13 shows the temperature distribution on the center of the copper-cored electrode at the peak time of the nth AC arc. The temperature near the end of the electrode is the highest, and the temperature decreases as it moves away from the end. With the increase in AC arc numbers, the arc continues to transport heat to the electrode, so the temperature on the electrode will also increase. As the arc current of the AC arc increases, the arc will also produce more heat, so the temperature on the electrode will also increase. Since the maximum temperature of the AC arc near the anode is higher than the maximum temperature near the cathode, the anode electrode will also transport more heat, resulting in a slight difference in temperature between the two electrodes.

## Conclusions

This paper proposes an AC arc experimental platform, and the number of AC arcs and arc current can be controlled. Subsequently, a two-dimensional axisymmetric MHD numerical simulation model of AC arc plasma is established. The voltage and current of the AC arc obtained by the AC arc MHD model have the same 'zero current' characteristics as the real AC arc in the experiment. Therefore, the heat generation error between the MHD model and the real arc is reduced. When the AC arc burns stably, the heat accumulates rapidly and heats up. When entering the 'zero current' stage, the temperature is reduced. The volume of the area with a temperature higher than 10,000 K increases with the arc current, but is unrelated to the number of AC arcs. The volume of the area with a temperature higher than 524.15 K increases with the number of AC arcs and the arc current. The temperatures on the electrode are positively correlated with the number of AC arcs and arc current.

## Data Availability

The datasets used and/or analyzed during the current study are available from the corresponding author upon reasonable request.
